# The Beneficial Role of Probiotic *Lactobacillus* in Respiratory Diseases

**DOI:** 10.3389/fimmu.2022.908010

**Published:** 2022-05-31

**Authors:** Tingfeng Du, Aihua Lei, Naiyu Zhang, Cuiming Zhu

**Affiliations:** Institute of Pathogenic Biology, Hengyang Medical College, University of South China, Hunan Provincial Key Laboratory for Special Pathogens Prevention and Control, Hengyang, China

**Keywords:** *Lactobacillus*, respiratory diseases, the gut-lung axis, mucosal immunity, safety

## Abstract

Respiratory diseases cause a high incidence and mortality worldwide. As a natural immunobiotic, *Lactobacillus* has excellent immunomodulatory ability. Administration of some *Lactobacillus* species can alleviate the symptoms of respiratory diseases such as respiratory tract infections, asthma, lung cancer and cystic fibrosis in animal studies and clinical trials. The beneficial effect of *Lactobacillus* on the respiratory tract is strain dependent. Moreover, the efficacy of *Lactobacillus* may be affected by many factors, such as bacteria dose, timing and host background. Here, we summarized the beneficial effect of administered *Lactobacillus* on common respiratory diseases with a focus on the mechanism and safety of *Lactobacillus* in regulating respiratory immunity.

## 1 Introduction

Probiotics are defined as “live microorganisms” and confer a health benefit on the host when properly administered (1). Many probiotics have been shown the beneficial properties, including *Lactobacillus* spp., *Bifidobacterium* spp., *Enterococcus* spp., *Streptococcus* spp., *Propionibacterium* spp., *Bacillus cereus*, *Saccharomyces boulardii*, and several specific strains of *Escherichia coli* (1, 2). Among probiotics, the most widely used are microorganisms of the genus *Lactobacillus*, which contains more than 200 species bacteria (3, 4). *Lactobacillus* spp. are gram-positive, facultative anaerobic bacterium that ferments carbohydrates to produce lactic acid (5), and has a high economic value due to the use in biotechnology, food as well as therapeutic application (4, 6, 7). In human hosts, *Lactobacillus* spp. exist in the gastrointestinal tract, vagina, oral cavity, respiratory tract and skin. They account for 6% and 95% of the total bacteria in the intestinal and vaginal tracts, respectively (8).

As natural immunobiotics, *Lactobacilli* have excellent immunomodulatory function, and their ability in improving gastrointestinal, oral and vaginal disorders is already well known (6, 9–12). Meanwhile, emerging evidence shows that *Lactobacillus* can also modulate respiratory immunity (13–16). Administration of *Lactobacillus* confer a beneficial role in respiratory diseases including respiratory tract infections (RTIs), asthma, lung cancer, cystic fibrosis (CF) and chronic obstructive pulmonary disease (COPD) (17–21). Administration of *Lactobacillus* may be an alternative strategy to alleviate respiratory diseases. In this review, we focus on the beneficial effect of probiotic *Lactobacillus* in promoting respiratory health and discuss its potential mechanism and safety.

## 2 Beneficial Role of *Lactobacillus* in Respiratory Diseases

### 2.1 Respiratory Tract Infections

RTIs are the major source of incidence and mortality in the world (22). Although many RTIs are of mild and self-limited nature, they caused 4 million deaths worldwide each year (23, 24). In 2017, influenza virus has infected 54.5 million people worldwide, resulting in about 145,000 deaths (25). Severe Acute Respiratory Syndrome Coronavirus-2 (SARS-CoV-2) is raging globally. Effective vaccines are not available for many respiratory pathogens, and the increase in drug-resistant microbe makes the effective treatment of RTIs extremely challenging. Therefore, it is important to find a safe and effective method to reduce the risk of RTIs. Recently, the probiotic *Lactobacillus* has been used to fight against a variety of RTIs, including virus and bacterial infection **(** **Table 1** **)**. Oral administration of *Lactobacillus* can improve symptoms caused by poly(I:C) treatment (49). Many *Lactobacillus* species can prevent influenza virus infection, including *L. rhamnosus* GG, *L. casei* Shirota, *L. plantarum* DK119, *L. paracasei* MCC1849, *L. gasseri* SBT2055, *L. fermentum* CJl-112 and *L. kunkeei* YB38 (34, 50–55). Moreover, *Lactobacilli* also have excellent role as a vaccine or adjuvant in preventing influenza virus infection due to its safety and biotechnological advantage (56–58). It is worth pointing out that oral supplementation with *L. johnsonii* (strain not shown) in pregnant BALB/c mice can reduce Th2 type cytokines and lung inflammation in Respiratory Syncytial Virus (RSV)-infected newborn mice (59). It means *Lactobacillus* may have preventive effect for RTIs of offspring when administered to the mother. Notably, supplementation with live and inactivated bacteria of the same *Lactobacillus* strain (such as *L. rhamnosus* CRL1505) generally have similar effect (39, 40), suggesting that viability is not necessary for *Lactobacillus* achieve the protective immunoregulatory effect. However, nasally administration of,viable but not heat-killed *L. rhamnosus* CRL1506 can provide complete protection against RSV infection in mice (28). Although the reason needs further investigation, it shows that *Lactobacilli* efficacy is strain-dependent. Therefore, the selection of a *Lactobacillus* strain with potent immunomodulatory ability is crucial.

**Table 1:** Pre-clinical studies on the administration of the *Lactobacillus* for protection against bacterial and viral respiratory tract infections.

*Lactobacillus* strain	Pathogen	Dose androute ofadministration	Experimentalmodel	Benefits	References
*L. rhamnosus* CRL1505 and *L. johnsonii*	Respiratory syncytial virus	1 ×10^8^ CFU viable *L. rhamnosus* CRL1505, via oral 1 ×10^7^ CFU viable *L. johnsonii*, via intranasal	Infant BALB/c mice	Pulmonary viral load and injury are reduced	(26, 27)
*L. rhamnosus* CRL1505 and L. rhamnosus CRL1506	Viral pathogen molecular pattern poly(I:C) + Respiratory syncytial virus	1 ×10^8^ CFU L. rhamnosus CRL1505 or *L. rhamnosus* CRL1506, via intranasal	Female 3-week-old BALB/c mice	Pulmonary viral load and injury are reduced	28
*L. plantarum* NCIMB 8826 or *L. reuteri F275*	Pneumonia virus	1 ×10^9^ CFU viable L. plantarum NCIMB 8826 or L. reuteri F275, via intranasal	Wild-type BALB/c and C57BL/6 mice	Improvement in survival rate and reduction in lung viral load, pulmonary inflammation was reduced	(3)
*L. rhamnosus* GG (LGG)	Influenza virus H1N1 strain PR8	1 ×10^8^ CFU viable LGG or 200 µg heat-killed LGG, via intranasal	Infant C57BL/6 mice or seven-week-old female BALB/c mice	Improvement in survival rate and reduction in lung Inflammation	(29, 30)
*L. casei Shirota*	Influenza A/PR/8/34 (PR8, H1N1) virus	200 µg heat-killed *L. casei Shirota*, via intranasal	BALB/c female mice	Improvement in survival rate and reduction in lung viral load	(31)
*L. plantarum* 06CC2 and L. gasseri TMC0356	IFV A/PR/8/34(H1N1)	20 mg Lyophilized *L. plantarum* 06CC2 powder, via oral 10 mg lyophilized L. gasseri TMC0356, via oral	SPF female BALB/c mice (4 or 6-week-old)	Weight loss is suppressed, a survival rate is raised, pulmonary viral load is reduced	(32, 33)
*L. fermentum* CJL-112 and *L. kunkeei* YB38	Influenza A/NWS/33 (H1N1) virus	1 ×10^8^ CFU viable *L. fermentum* CJL-112, via intranasal 100 mg/kg heat-killed *L. kunkeei* YB38, via oral	Female, specific pathogen-free (SPF) BALB/c mice	Significant up-regulation of Th1cytokine and IgA and specific anti-influenza IgA levels Improvement in survival rate and reduction in pulmonary inflammation	(34)
*L. plantarum* nF1	Influenza A (H1N1 and H3N2 subtypes) and influenza B (Yamagata lineage) viruses	110 mg heat-killed *L. plantarum* nF1, via oral	BALB/c mice(5-week-old females)	Weight loss is suppressed and pulmonary viral load is reduced	(35)
*L. paracasei* CNCM I-1518	Influenza A(H3N2)	2 ×10^8^ CFU viable *L. paracasei* CNCM I-1518, via oral	Six-week-old female BALB/c mice	Weight loss is suppressed, pulmonary viral load and inflammation are reduced	(36)
*L. fermentum* CJL-112	Influenza A(H9N2) virus	1.5 ×10^9^ CFU viable *L. fermentum* CJL-112, via intranasal	Chicken	Improvement in survival rate	(37)
*L. paracasei* ST11	Vaccinia virus	1 ×10^8^ CFU viable *L. paracasei* ST11, via oral	Seven-weeks male Balb/c mice	Reduction in viral spread with a significant decrease of VACV titer on lung, liver and brain, lung inflammation is attenuated and survival rate is increased	(38)
*L. rhamnosus* CRL1505	Streptococcus *pneumoniae*	1 ×10^8^ CFU viable or non-viable *L. rhamnosus* CRL1505, via intranasa 8 µg peptidoglycan of *L. rhamnosus* CRL1505, via intranasal	Immunodeficient Swiss-albino mice	Lung load of pathogens and injury are reduced Improvement in survival rate	(39–41)
*L. pentosus* b240	S. *pneumoniae*	500 mg kg^-1^ heat-killed *L. pentosus* b240, via oral	Five-week-old male mice	Prolonged survival time, less body weight loss and lung viral load	(42)
*L. casei* CRL 431	Sd *pneumoniae*	1 ×10^9^ CFU viable *L. casei* CRL 431, via oral or via intranasal	Adult 8-week-old Swiss albino mice and immunodeficient Swiss-albino mice	Lung bacterial load is decreased and lung inflammation is reduced, accelerated weight recovery	(43, 44)
*L. casei* CRL 431 and LGG	Pseudomonas *aeruginosa*	1 ×10^9^ CFU viable L. casei CRL 431, via oral 1 ×10^9^ CFU viable LGG, via oral	Three-week-old mice (young mice)	Bacterial clearance of lung tissue is increased Improvement in survival rate and reduction in lung Inflammation	(45, 46)
*L. plantarum* CIRM653	*Klebsiella pneumoniae*	1 ×10^8^ CFU viable *L. plantarum* CIRM653, via oral	6-8-week-old C57/BL6J mice	The pulmonary inflammation response is reduced	(47)
*L. murinus* CNCM I-5314	*Mycobacterium tuberculosis* (H37Rv)	1 ×10^7^ CFU viable L. *murinus*, via oral	Six-eight-week-old female SPF C57BL/6 mice	reduction in pulmonary inflammation	(48)

Although vaccines for SARS-CoV-2 are available, the rapidly SARS-CoV-2 mutating makes the effectiveness of these vaccines challenging (60). Notably, *in vitro* experiment has demonstrated that *L. fermentum* 90 TC-4 pretreatment increases the activity of SARS-CoV-2-infected Vero E6 cells (grass monkey kidney cells) (61). Pretreatment *of L. plantarum* MPL16 and CRL1506 can also inhibit the proliferation of SARS-CoV-2 in human lung epithelial cell line Calu-3 (62). In Central Europe, it has been reported that low death rate of COVID-19 patients is associated with the consumption of fermented vegetables containing many *Lactobacilli* species (63). Therefore, *Lactobacilli* may have the potential to become an adjuvant for treating SARS-CoV-2.

However, there are some limitations in the therapeutic effect of administering *Lactobacillus*. For example, neonatal C57BL/6 mice pre-treated with *L. rhamnosus GG* maintain 100% survival rate post influenza virus infection; however, the survival rate is only 10% when mice treated with *L. rhamnosus GG* at 48h post influenza virus infection (29). Similarly, the survival rate of BALB/c mice treated with *L. plantarum* at 24h post Pneumonia virus infection is 100%, but all mice die when *L. plantarum* administered at 72h post infection (3, 64). These results show that *Lactobacillus* administration post infection cannot exert an obvious protective immunoregulatory effect.

*Lactobacilli* can also provide resistance to respiratory bacterial infections. Administration of *Lactobacilli* such as *L. rhamnosus* CRL1505, *L. casei* CRL 431 and *L. pentosus* B240 increases resistance of mice to *Streptococcus pneumoniae* infection (41–43). Intranasal inoculation of *L. rhamnosus* CRL1505 is beneficial in *S. pneumoniae* infected-immunodeficient mice (39). Interestingly, peptidoglycan from *L. rhamnosus* CRL1505 shows a similar protective effect with the whole bacteria in preventing *S. pneumoniae* infection (40). Moreover, nasal administration of *L. rhamnosus* CRL1505 can also reduce pathogen load and lung damage of infant mice with RSV infection and secondary *S. pneumoniae* infection (65, 66).

In clinical trials, *Lactobacilli* are generally given in the form of tablets, capsules, powders, fermented yogurt or dairy products, and mainly used for preventive purposes (**Table 2**). For instance, oral *L. rhamnosus* GG in adults can reduce rhinovirus infection (83), and the combination of oral *L. paracasei* (strain not shown), *L. casei* CRL 431 and *L. fermentium* PCC also reduces rhinovirus-induced common and influenza-like infection (78). In addition, oral mixed probiotic (mainly *Lactobacilli*) can decrease the risk of respiratory failure in COVID-19 patients by 8-fold and reduce the rate of transfer to Intensive Care Unit and mortality (84). In patients with severe COVID-19, oral tablet (live *B. longum*, live *L. bulgaricus* and live *S. thermophilus*, strains not shown) also shortens the time to reach a negative nucleic acid test of SARS-CoV-2 and decreases blood C-reactive protein and procalcitonin (81). Moreover, clinically administration of many *Lactobacillus* species such as *L. rhamnosus* GG, *L. paracasei* N1115 and *L. plantarum* L-137 can decrease the total incidence as well as shorten the duration of RTIs (26, 27, 81). Therefore, the prospect of *Lactobacillus* clinical application in reducing the risk of RTIs is promising.

**Table 2:** Treatment effect of clinical trials regarding the application of *Lactobacillus* in improving symptoms of respiratory tract infections (RTIs).

Lactobacillus strain	Subjects	Efficacy	References
*L. rhamnosus* GG	Premature infants	The reduction in the incidence of RTIs	(67)
*L. casei Shirota*	Healthy middle-aged working people	Reducing the incidence and duration of upper respiratory tract infections (URTIs)	(68)
*L. casei* DN 114001	Healthy school-age children	The reduction in the incidence and duration of RTIs	(69)
*L. reuteri* SD 112	Infants	Reducing the rate and duration of RTIs	(70)
*L. plantarum* L-137	Healthy subjects with high psychological stress	The reduction in the incidence of URTIs	(71)
*L. plantarum* DR7	Adults	Improving clinical symptoms of URTIs	(14)
*L. paracasei* N1115	Older Adults	Strengthening resistance of RTIs	(72)
*L. fermentum* CECT5716	Infants	Reducing the incidence of URTIs	(73)
*L. fermentum* PCC	Athletes	The reduction in lower respiratory symptoms in men	(74)
*L. salivarius*	Athletes	No effect on the frequency of URTIs	(75)
*L. helveticus* Lafti L10	Athletes	Shortening the duration of RTIs	(76)
Combination of *L. rhamnosus* GG, *L. rhamnosus* LC705, B. *breve* 99, *P. freudenreichii* JS	Children	Reducing the incidence of RTIs	(77)
Combination of *L. paracasei*, *L. casei* 431, *L. fermentium* PCC	Adults	Strengthening resistance of common cold and flu-like respiratory infections	(78)
Combination of *L. acidophilus*, *B. lactis* UABLA-12	Children	Do not reduce the incidence, but shorten the duration of acute respiratory infections	(79)
Combination of *L. gasseri* PA16/8, B. longum SP07/3	Adults	Reducing the duration of RTI episodes and fevers	(80)
Combination of *B. longum*, *L. bulgaricus* and *S. thermophilus*	Patients with COVID-19	The duration of diarrhea is shortened. Significantly shorter time to nucleic acid negativity and significantly lower inflammatory markers such as calcitoninogen and C-reactive protein	(81)
Combination of *L. plantarum* KABP022, KABP023, KAPB033, and *P. acidilactici* KABP021	Patients with COVID-19	The reduction in nasopharyngeal viral load, pulmonary infiltration, and duration of digestive and non-digestive symptoms.	(82)

### 2.2 Asthma

Asthma is a heterogeneous airway disease, which behaves as complex symptoms, including cough, intermittent wheezing, dyspnea, chest tightness, airway obstruction, and bronchial hyperresponsiveness. Asthma patients generally have a Th1/Th2 imbalance and are polarized toward Th2 type immune response, usually resulting in high level of allergen-specific IgE and eosinophilic airway inflammation (85). Although asthma can be treated by strategies include allergen avoidance and improvement of signs and symptoms by inhaled corticosteroids, anti-leukotrienes and β2 agonists, there is still no specific treatment for asthma and healthcare is expensive during exacerbation (86).

Emerging evidence suggests that respiratory asthma symptoms can be ameliorated when probiotic *Lactobacillus* is administered. Oral administration of many *Lactobacillus* species has shown an effective preventive role for asthma in animal studies, including *L. rhamnosus* GG, *L. plantarum* K37, *L. reuteri* (ATCC No. 23272), *L. casei* Shirota, *L. paracasei* HB89 and *L. salivarius* (strain not shown) (18, 87–91). Some *Lactobacillus* species such as *L. rhamnosus* GG, *L. paracasei* (strain not shown) and *L. fermentum* (strain not shown) are orally used in the clinical and have beneficial effect on asthma in children (92, 93). Interestingly, the acute asthma is closely associated with RTIs, especially rhinovirus infection (94). Consequently, the role of *Lactobacillus* administration in preventing RTIs may be useful in relieving exacerbation of acute asthma. Based on the “hygiene hypothesis,” exposure to specific microbial components early in life can decrease the susceptibility to asthma and allergic diseases (95). Therefore, oral administration of probiotic *Lactobacillus* in the early life may also play an important role in preventing asthma.

For the purpose of asthma prevention, the general route of administration of *Lactobacillus* species is oral delivery. However, a study indicates that although oral administration of *L. paracasei* NCC2461 could provide effective protection for female BALB/c mice with asthma, the efficacy of intranasal delivery is better (96). Nevertheless, two studies show that oral administration of *L. rhamnosus* GR-1 prevents the worsening of asthma in male BALB/c mice, but nasal administration has little effect on the improvement of asthma symptoms in male BALB/c mice (97, 98). This may be caused by the lower dose and duration of nasal administration. Interestingly, oral administration of *L. paracasei* NCC2461 in perinatal mice provides prevention for allergic airway inflammation in the offspring (99). Similarly, in clinical trials, oral *L. rhamnosus* GG has been shown to prevent atopic diseases in children in both prenatal and postnatal (100). Thus, *Lactobacillus* may have a preventive effect against offspring asthma when administered to the mother. Furthermore, supplementation with *Lactobacillus* GG also has a therapeutic rather than only a preventive effect on mice suffering from asthma (101). Additionally, animal studies have found that oral *Lactobacillus* can also reduce other airway allergic reactions such as allergic rhinitis. For example, oral *L. rhamnosus* GG, *L. gasseri* TMC0356, *L. plantarum* IM76, *L. plantarum* CJLP133 and CJLP243 can effectively improve the symptoms of allergic rhinitis (102–104). In clinical trials. *L. gasseri* KS-13, *L. casei* Shirota and *L. acidophilus* L-92 have been used to effectively prevent seasonal allergic rhinitis (105–107). Thus, the administration of *Lactobacillus* may have excellent preventive effect on airway allergic reaction.

### 2.3 Lung Cancer

Lung cancer is a malignant tumor with a high incidence and mortality rate in the world (108). In recent years, immunotherapy has sparked a new surge in tumor treatment. Although immune check inhibitors (ICIs) such as anti-PD-1/PD-L1 and anti-CTLA 4 antibodies has become the first-line clinical treatment for tumors, the application of ICIs increases T-cell activity and removes the “braking” of the immune system, and these drugs are likely been associated with immune-related adverse events, especially when used in combination (109). Therefore, the search for natural immunobiotics with immunomodulatory properties to assist in the treatment of cancer patients may be a direction. It has been reported that probiotics show the same degree of anti-tumor ability as PD-L1 inhibitors, while simultaneous combination therapy with PD-L1 inhibitors virtually eliminated tumor growth in mice (110). Moreover, in clinical trials, oral administration of yogurt with probiotics provides a potential protective effect against lung cancer (111). *Lactobacillus* is one of the most widely studied probiotic involved in the treatment of lung cancer. Administration of *Lactobacillus* can inhibit the metastasis of tumor cells to the lung. For example, nasal administration of *L. rhamnosus* GG can inhibit the metastasis of melanoma B16 to the lung in C57BL/6 mice (112). *L. casei* (strain not shown) has significant inhibitory effect on the proliferation of A549 lung cancer cells *in vitro* (113). Furthermore, intravenous and intradermal injection of *L. casei* YIT 9018 can increase the anti-tumor activity against Lewis lung carcinoma in C57BL/6 mice (114). In addition, oral administration of *L. casei* CRL 431 also reduces the side effects of chemotherapy (115), which may improve the prognosis of lung cancer patients. These suggest that some *Lactobacillus* species may have potential to become an effective adjuvant to treat lung cancer.

### 2.4 CF

CF is an autosomal recessive, monogenic disease with lesions affecting the lung, intestine, and other organ, but lung disease is the leading cause of morbidity and mortality in people with CF (116). CF is closely related to intestinal flora and significantly reduces intestinal flora diversity (117, 118). A study indicates that oral *L. rhamnosus* GG improves disorders of intestinal flora in children with CF (119). Moreover, clinical trial shows oral administration of *L. rhamnosus* GG reduces pulmonary exacerbation and hospitalization rate in CF patients (120). This suggests administration of *L. rhamnosus* GG may be able to alleviate the symptoms of CF. In another clinical trial, however, oral administration of *L. rhamnosus* GG did not promote respiratory health in patients of CF (121). The main reason may be due to different inclusion criteria for clinical trial subjects. The clinical efficacy of applying a single *Lactobacillus* strain on CF is unstable, and the combined administration of multiple probiotic *Lactobacillus* species may be more effective. For example, intestinal supplementation with probiotic capsules (consisting mainly of *Lactobacillus* species) significantly reduces the incidence of lung deterioration and improves the quality of life in patients with CF (19, 122). However, these are single-center clinical trials. Multicenter and expansion of the number of subjects are needed to further clarify the efficacy of *Lactobacillus* administration on CF in clinical trials.

### 2.5 Other Respiratory Diseases

Several studies have reported the potential of *Lactobacillus* in improving COPD. The commonly known causes of COPD are cigarettes and air pollutants (123). *In vitro* experiment has demonstrated that *L. rhamnosus* NutRes1 can reduce inflammatory mediators produced by cigarette-activated human macrophages (124). Moreover, oral feeding of *L. rhamnosus* (strain not shown) increases levels of IL-10 as well as SOCS3 and TIMP1/2, and attenuates lung injury of COPD induced by cigarette in C57BL/6 mice (21). Additionally, the development of COPD is associated with reduction of *Lactobacillus* spp. in lung (125).

In addition, a study indicates that a decrease in intestinal *Lactobacillus* may contribute to the development of pulmonary arterial hypertension (126). In clinical trial, intestinal supplementation with *L. casei* Shirota can reduce the incidence of Ventilator-Associated Pneumonia in hospitalized patients (127). Thus, *Lactobacillus* may also be beneficial in other respiratory diseases and need more studies.

## 3 Potential Mechanism of Probiotic *Lactobacillus* in Improving Respiratory Diseases

As mentioned above, many members of the genus *Lactobacillus* have immunomodulatory properties. Notably, not only the whole bacteria, but also their components can exert immunomodulatory function, including peptidoglycans, extracellular polysaccharides, surface proteins and metabolites (short-chain fatty acids, SCFAs) and inorganic polyphosphate liquids (40, 128–131). The mechanism of health-promoting effect of *Lactobacillus* on respiratory tract is complex. The same bacteria, when administered orally or nasally, or even administered live and inactivated bacteria, may not have the same effect on promoting respiratory health (49, 50, 52). The potential mechanisms include gut-lung axis and enhancement of the local mucosal immunity of the respiratory tract.

### 3.1 The Gut-Lung Axis

**Figure 1:**
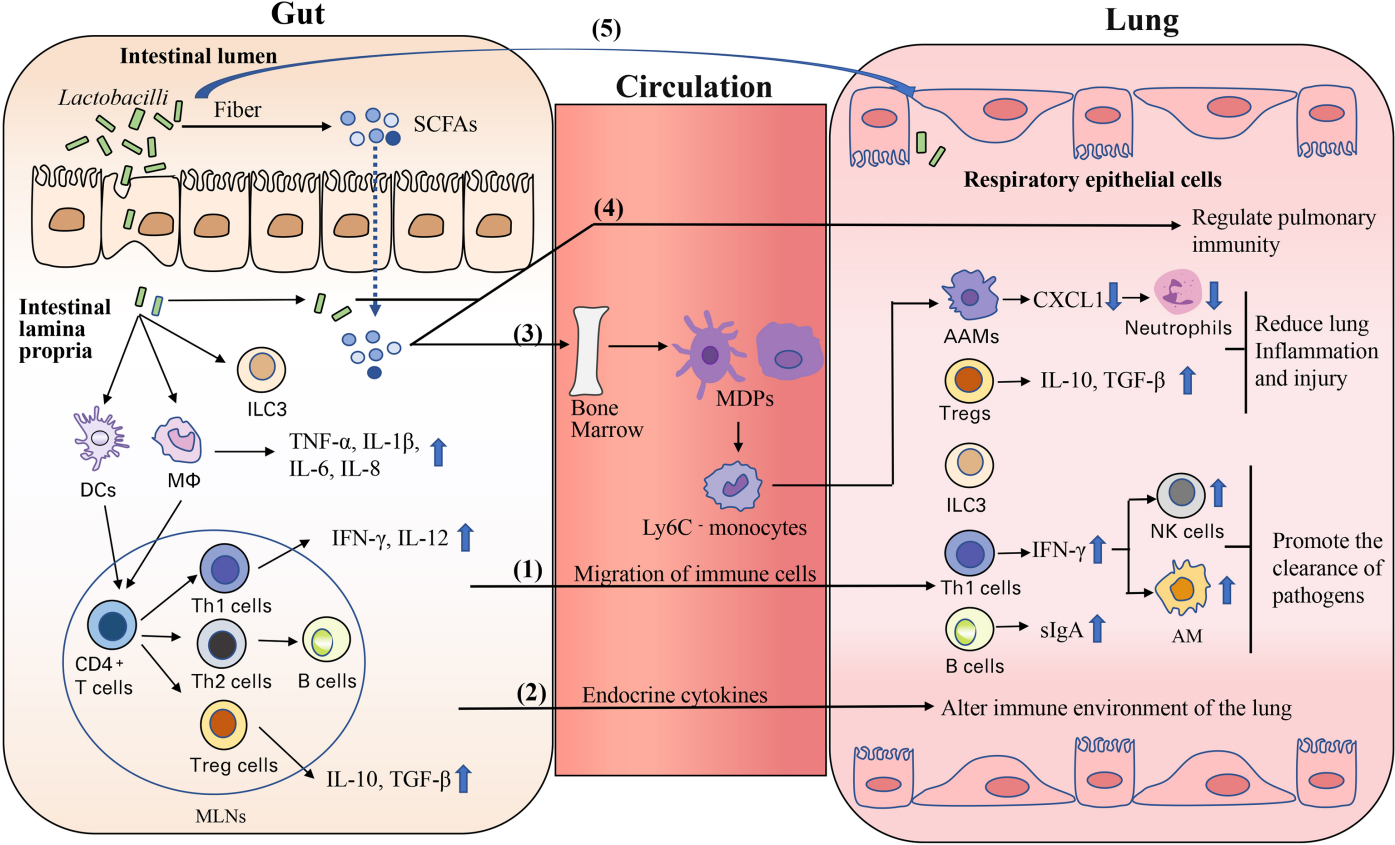
Potential mechanisms of *Lactobacilli* to modulate respiratory immunity *via* the gut-lung axis (1). Migration of activated immune cells and cytokines from mesenteric lymph nodes (MLNs) and intestinal lamina propria to the lung through the circulation (2). Some endocrine cytokines (such as TNF-α, IL-6) may migrate to the lung tissue through the circulation, and then alter immune environment of the lung (3). SCFAs affect bone marrow hematopoiesis and promote the conversion of macrophage and DC progenitors (MDPs) into Ly6C-monocytes, which reaches lung tissue and differentiates into anti-inflammatory alternatively activated macrophages (AAMs); AAMs inhibit chemokine CXCL1 production thus leading to reduced neutrophils recruitment in lung tissue (4). In the intestinal lumen, *Lactobacilli* or their components and production of metabolites (such as SCFAs) are taken up by intestinal epithelial cells and then enter to the lung *via* the circulation (5). *Lactobacilli* or their components from the intestinal lumen reach lung directly *via* microbreathing or esophageal reflux.

In recent years, many studies have reported that there is a crosstalk between gut and lung, and this connection described as gut-lung axis seems to be bidirectional (132, 133). Oral administration of some probiotics, especially *Lactobacilli*, could promote respiratory health *via* gut-lung axis (17, 30, 134). Although the exact mechanisms by which *Lactobacillus* enterica affects the lung immunity *via* gut-lung axis are not fully understood, there are three main aspects (**Figure 1**).

#### 3.1.1 Direct Immigration of Immune Cells and Cytokines From the Intestine to the Respiratory Tract Through the Circulation

Recognition of *Lactobacillus* species or their components by pattern recognition receptors (PRRs, such as TLRs or NLRs, etc.) on immune cells in the intestinal mucosa can result in the activation of innate immune cells which could migrate to lung tissue *via* circulation. For instance, innate lymphoid group 3 cells (ILC3s) can migrate from the gut to the lung where IL-22^+^ILC3 exhibits resistance to pneumonia (135). Oral delivery of *L. paracasei* CNCM I-1518 also significantly increases ILC3 in lung tissue and enhances resistance to influenza virus (36). Intestinal supplements with *L. paracasei* MCC1849 can provide protection against influenza virus, which may be associated with an increased proportion of IgA^+^ B cells and follicular helper T cells in Peyer’s patches and significantly increases IgA secretion in lung (53). Villena suggests that Oral delivery of *L. rhamnosus* CRL1505 induces Th1 response in the gut-associated lymphoid tissue and promotes the migration of Th1 cell to the lung tissue where Th1 cells produces IFN-γ which induces activation of alveolar macrophages, natural killer cells, CD103^+^ and CD11b^high^ dendritic cell (DC) (49). Additionally, a study indicates that intestinal supplementation with *L. rhamnosus* GG can inhibit pulmonary inflammation, which is associated with an increase in Tregs of the intestinal tract (136). Moreover, oral administration of *L. murinus* (strain not shown) to antibiotic-treated mice can also increase Tregs in lung (137). Tregs not only inhibit inflammation, but also inhibit Th2 type immune response (138). As controlling airway inflammation is crucial in preventing asthma progression (94), migration of Tregs to lung tissue may alleviate asthma symptoms.

Cytokines secreted in the mucosa of the gastrointestinal tract by *Lactobacillus* can reach the circulation and interact with other mucosal tissues, such as lung (139). Intestinal supplementation with *L. rhamnosus* CRL1505 can increase TNF-α, IFN-α, IFN-β, IFN-γ in bronchoalveolar lavage fluid which exert a significant antiviral effect in the respiratory tract, and these cytokines show a similar increased trend in the intestinal fluid (49).

#### 3.1.2 Influence of *Lactobacillus* Metabolites on Pulmonary Immunity *via* the Circulation

In parallel to promoting the migration of intestinal immune cells and cytokines, members of *Lactobacillus* genus produce metabolites that can modulate host respiratory immunity. The most important intestinal bacteria metabolites affecting pulmonary immunity is SCFAs, such as propionate, butyrate and acetate (140). Studies show that intestinal supplementation with many *Lactobacillus* species can increase SCFAs in the intestine and blood (141–145). The unmetabolized SCFAs enter the circulation and may affect pulmonary immunity in two ways. The first way is that SCFAs enter the bone marrow through the circulation enhancing generation of macrophage and DC progenitors (MDPs) (146), and meanwhile SCFAs can increase the conversion of MDPs into Ly6C^–^ monocytes (147, 148); then bone marrow Ly6C^–^ monocytes migrate into lung tissue where they differentiate into alternatively activated macrophages (149). The second way is that SCFAs directly enter into the lung tissue where they may activate G protein-coupled receptors (GPCRs) or inhibit histone deacetylase (150–152). For example, butyrate can not only promote the generation of Tregs and IL-10 production by activating GPR109A (153), but also restore IL-10 in the lung by inhibiting histone deacetylase in mice (154). Another metabolite of *Lactobacillus* enterica, lithocholic acid, can also enhance Tregs function while inhibit Th17 response (155). Other *Lactobacillus* intestinal metabolites (such as lactic acids, polyamines and indole derivatives) with immunomodulatory properties are involved in intestinal homeostasis (156–158). Nevertheless, it remains to be studied whether these metabolites can impact respiratory health *via* the gut-lung axis. In summary, *Lactobacillus* metabolites, such as SCFAs (especially butyrate) and lithocholic acid have excellent anti-inflammatory capacity, which may contribute to alleviate the development of RTIs, asthma, COPD. In addition to anti-inflammatory effect, butyrate and propionate can induce apoptosis of lung cancer cells and inhibit proliferation of lung cancer cells *in vitro* (159–161). Therefore, oral administration of some *lactobacilli* may provide benefit for the treatment of lung cancer by producing butyrate and propionate.

#### 3.1.3 Migration of *Lactobacillus* and Their Components to the Lung

There may be two main routes for transfer of intestinal bacteria to the lung tissue: the first is intestinal bacteria or bacterial components enter into the circulation through the mesenteric lymphatic system and then reach the lung tissue (162–164); the second is the migration of intestinal bacteria or bacterial components to the lung tissue through microbreathing and oropharyngeal reflux (165, 166). Therefore, *Lactobacillus* or components of *Lactobacillus* in the intestine may be directly transferred to lung tissue and thus modulate lung immunity.

### 3.2 Enhancement of the Mucosal Immunity of the Respiratory Tract

Intestinal supplementation with *Lactobacillus* has been shown to promote respiratory health, but direct action of immunobiotic *Lactobacillus* on the respiratory mucosa may modulate local immunity of the respiratory tract. It has been found that intranasal administration of *Lactobacillus* can induce better respiratory immune response than oral administration (3, 50, 167). Nasal administration of *Lactobacillus* does not generally produce SCFAs due to the absence of substrate. The potential mechanisms by which they regulate respiratory immunity mainly have two aspects.

The first one is that some components of *Lactobacillus* can be recognized by PRRs in the respiratory tract and then activate downstream pathways. For example, nasal priming with peptidoglycan from *L. rhamnosus* CRL1505 increases TNF-α and IL-10 levels of lung and upregulates TLR2 and TLR9 expression in alveolar macrophages, which is similar to intranasal administration of whole bacteria (40). Meanwhile, other studies have shown that nasal priming with peptidoglycan from *L. rhamnosus* CRL1505 can enhance the TLR3/RIG-I-triggered antiviral immune response by increasing IFN-γ and NK cell activity, thus contributing to higher viral clearance and reducing lung tissue damage (28, 65, 168). In addition, lung peptidoglycan can also be recognized by peptidoglycan recognition proteins (PGRPs), a type of PRRs, which mediates bactericidal effect (169). For instance, activated PGRP2 could promote neutrophil recruitment in lung tissue of *S. pneumoniae* infected mice (170). Of note, not all peptidoglycan of *Lactobacillus* species has the same protective effect. Nasal administration the peptidoglycan from *L. rhamnosus* CRL534 does not enhance resistance to *S. pneumoniae* infection in immunodeficient mice (41). This strongly suggests that the protective effect provided by *Lactobacillus* is strain-specific. Importantly, nasal administration of *Lactobacillus* may activate PRRs by multiple pathways. Even if one PRR is blocked, another pathway can be activated to provide protection in a compensatory manner. For example, studies have shown that only when both NOD2 and TLR2 are knocked out can *L. plantarum* BAA-793 lose its role in protecting against pneumonia virus infection (3, 64, 171). Therefore, components of *Lactobacillus* to activate the PRRs may be an important part of the protective role performed by intranasal *Lactobacillus* delivery.

The second one is that *Lactobacillus* can bind to host cells to antagonize adhesion or binding of pathogen. For the bacteria, experiments have demonstrated the ability of *Lactobacillus* to directly inhibit the adhesion of bacteria to respiratory epithelial cells. *L. rhamnosus* Kx151A1, *L. reuteri* PTA-5289, and *L. salivarius* LMG9477 can inhibit the adhesion of *S. pyogenes* to pharyngeal epithelial cells (172). Moreover, intranasal administration of *L. murinus* CNCM I-5314, a eubacterium of the murine lung, can provide a barrier function against the colonization of *S. pneumoniae* in the lung tissue (173). In the case of viruses, *Lactobacillus* binds competitively to viral receptor molecule to prevent viral entry into the host cell. For instance, lipopeptides released by *L. curvatus*, *L. sakei* and *L. lactis* (strains not shown) can bind to the receptor molecule (angiotensin-converting enzyme 2) of SARS-CoV-2 spike glycoprotein, and may prevent virus entry into host cells (174, 175). In addition to inhibiting the adhesion and binding of pathogenic bacteria, *Lactobacillus* directly displays antibacterial activity. Some *Lactobacillus* spp. exhibit antibacterial effect against group A *Streptococcus in vitro* (176). Similarly, *L. rhamnosus* Kx151A1 *and L. reuteri* PTA-5289 significantly inhibit hemolytic activity of *S. pyogenes in vitro* (172). Additionally, some proteins secreted by *Lactobacillus* have antimicrobial activity. For example, reuterin secreted by *L. reuteri* has broad-spectrum antibacterial effect (2). However, whether it will alter lung microbial composition and affect lung homeostasis remains further investigation.

## 4 Safety

As normal members of the human intestinal, vaginal, skin, oral and respiratory flora, *Lactobacilli* are low-toxicity commensal organisms and are mostly considered safe when taken as probiotics. In animal studies, long-term oral administration of *L. plantarum* PS128 had no bad side on the health in mice (177). In addition, nasal inoculation of *L. reuteri* F275 and *L. rhamnosus* GG, which are generally colonized in the intestinal tract. *L. reuteri* F275 is cleared in lung tissue less than 24 hours (178); live *L. rhamnosus* GG is detected on the nasal mucosa at 24 hours after intranasal administration, but not after 72 hours, and does not affect body weight or behavior in mice (97). In clinical trials, oral administration of some common *Lactobacillus* species such as *L. reuteri* DSM17938, *L. casei* Shirota and *L. salivarius* CECT5713 is safe in infants or children (2, 179–181). Moreover, with a long history of safe use, *Lactobacilli* are classified as GRAS (Generally Recognized as Safe) and QPS (Qualified Presumption of Safety) by the US Food and Drug Administration (FDA) and the European Food Safety Authority (EFSA, 2021), respectively (182). Therefore, administration of some *Lactobacillus* species is generally safe.

However, when *Lactobacillus* is given intranasally, it can colonize the respiratory tract for long periods of time, and whether this could interfere lung microbial homeostasis or induce more severe inflammation or even lead to bacteremia is not clear. Although *Lactobacillus* bacteremia is a rare disease, it has been found in the clinical that *L. rhamnosus* GG and *L. casei* (strain not shown), which are generally considered safe, can also cause bacteremia when in a state of immunosuppression, prolonged hospitalization or surgical intervention (183). Even non-pathogenic bacteria of *L. salivarius* (strain not shown) in the oral cavity have been found to cause bacteremia, septic chest and diabetic ketoacidosis due to respiratory failure in the clinical (184). Moreover, it has been reported that pneumonia and pleural abscess are caused by mixed *Lactobacillus* infection in elderly people with esophageal cancer (185). Although it occurs being rare and almost exclusively in infants, children and immune-compromised populations, this also suggests that live *Lactobacillus* is not absolutely safe, especially when administered intranasally. Further, the presence of antibiotic resistance genes and virulence genes in *Lactobacillus* and their possible transfer to other microorganisms is also a concern.

## 5 Concluding Remarks

The use of *Lactobacillus* is a promising strategy for the prevention and treatment of respiratory diseases and is generally safe. The mechanism of *Lactobacillus* in regulating respiratory immunity includes the gut-lung axis and activation of mucosal immunity. The beneficial role of *Lactobacillus* on the respiratory tract is strain-dependent, and may change in different species of *Lactobacillus* and even subspecies of each *Lactobacillus* (32). Therefore, for clinical applications, the selection of effective *Lactobacillus* strains is crucial. Additionally, a single *Lactobacillus* is not always effective for all respiratory diseases and efficacy may be inconsistent even if the same *Lactobacillus* is administered due to many factors such as subject, dose and time of administration in clinical trials. The uncertainty of the effectiveness of *Lactobacilli* is one of the main reasons limiting their application in the clinic. For this problem, co-administration of multiple probiotic *Lactobacillus* or co-administration of probiotic *Lactobacillus* and other probiotics such as *Bifidobacterium* can provide more stable and better efficacy, which is also the trend of probiotic application. Furthermore, clinical application of the main active ingredients of *Lactobacillus* or inactivated bacteria can provide more safer effect and stable efficacy relative to live bacteria. Besides, *Lactobacillus* has preventive effect for offspring respiratory disease when administered to the mother, and it can greatly reduce the occurrence and alleviate the symptoms of respiratory diseases in infants and children if the stability and safety of *Lactobacillus* efficacy is fully established. Additionally, the economic burden of clinical application of *Lactobacillus* is low (186). Overall, administration of *Lactobacillus* is beneficial in improving pulmonary health and its application in treating respiratory diseases needs more clinical studies.

## Author Contributions

TD finished the original manuscript. NZ compiled table information. CZ and AL provided constructive comments and made critical revisions to the manuscript. All authors contributed to the article and approved the submitted version.

## Funding

CZ was supported by National Natural Science Foundation of China (No. 31970177); AL was supported by the National Natural Science Foundation of Hunan Province (No. 2021JJ40475).

## Conflict of Interest

The authors declare that the research was conducted in the absence of any commercial or financial relationships that could be construed as a potential conflict of interest.

## Publisher’s Note

All claims expressed in this article are solely those of the authors and do not necessarily represent those of their affiliated organizations, or those of the publisher, the editors and the reviewers. Any product that may be evaluated in this article, or claim that may be made by its manufacturer, is not guaranteed or endorsed by the publisher.

## Abbreviations

RTIs, Respiratory infections; CF, Cystic fibrosis; COPD, Chronic obstructive pulmonary disease; RSV, Respiratory Syncytial Virus; SARS-CoV-2, Severe Acute Respiratory Syndrome Coronavirus-2; SCFAs, Short-chain fatty acids; DC, Dendritic cell; BALF, Bronchoalveolar lavage fluid; PRR, Pattern recognition receptor; TLRs, Toll-like receptors; NLRs, NOD-like receptors; Tregs, Regulatory T cells.
